# Impact of Cumulative Embryo Implantation Failures on Embryonic Ploidy Status and Post-PGT-A Clinical Outcomes: A Retrospective Cohort Analysis

**DOI:** 10.3390/genes17040389

**Published:** 2026-03-29

**Authors:** Jie Li, Wei Zhou, Tianxiang Ni, Yueting Zhu, Qian Zhang, Junhao Yan

**Affiliations:** 1 State Key Laboratory of Reproductive Medicine and Offspring Health, Center for Reproductive Medicine, Institute of Women, Children and Reproductive Health, Shandong University, Jinan 250012, China; lijie19992023@163.com (J.L.);; 2 National Research Center for Assisted Reproductive Technology and Reproductive Genetics, Shandong University, Jinan 250012, China; 3 Key Laboratory of Reproductive Endocrinology (Shandong University), Ministry of Education, Jinan 250012, China; 4 Shandong Technology Innovation Center for Reproductive Health, Jinan 250012, China; 5 Shandong Provincial Clinical Research Center for Reproductive Health, Jinan 250012, China; 6 Shandong Key Laboratory of Reproductive Research and Birth Defect Prevention (Under Construction), Jinan 250012, China; 7 Research Unit of Gametogenesis and Health of ART-Offspring, Chinese Academy of Medical Sciences (No. 2021RU001), Jinan 250012, China

**Keywords:** recurrent implantation failure, live birth, embryonic ploidy status, preimplantation genetic testing for aneuploidy, maternal age

## Abstract

**Objective:** To investigate the relationship between the number of previous implantation failures (IFs) and embryo ploidy status, as well as subsequent clinical outcomes, in women with recurrent implantation failure (RIF) undergoing preimplantation genetic testing for aneuploidy (PGT-A). **Methods:** This retrospective cohort study included 422 women with RIF who underwent their first PGT-A cycle between 2017 and 2022. Participants were stratified by maternal age (<38 years, n = 292; ≥38 years, n = 130) and by the number of previous IFs, categorized as 3, 4, or ≥5. The primary outcomes were embryo ploidy rates (euploidy, aneuploidy, and mosaicism). Secondary outcomes included reproductive outcomes after single euploid blastocyst transfer (biochemical pregnancy, clinical pregnancy, ongoing pregnancy, live birth, and pregnancy loss) and neonatal birth weight. **Results:** Women aged ≥38 years had a significantly lower euploidy rate than those <38 years (24.8% vs. 47.3%, *p* < 0.001). Ploidy distribution did not differ significantly across IF categories. Among women aged <38 years with ≥5 IFs, a greater number of previous embryo transfer attempts was independently associated with higher odds of live birth after euploid embryo transfer (adjusted OR = 1.258, 95% CI: 1.051–1.505; *p* = 0.012). Neonatal weight did not differ significantly across IF categories. **Conclusions:** The number of previous IFs was not independently associated with embryo ploidy or clinical outcomes after euploid transfer, whereas advanced maternal age was strongly associated with a lower likelihood of obtaining euploid embryos. In younger women with ≥5 IFs, a greater number of previous embryo transfer attempts was associated with live birth after euploid transfer; however, this exploratory subgroup finding should be interpreted cautiously and requires prospective validation. Because this study did not directly evaluate therapeutic strategies, any potential role for individualized endometrial evaluation or optimization should be considered as hypothesis-generating rather than supported by the present data.

## 1. Introduction

Despite ongoing advances in in vitro fertilization and embryo transfer (IVF-ET), a subset of patients still fails to achieve pregnancy after repeated transfers of high-quality embryos, a condition referred to as recurrent implantation failure (RIF) [1]. RIF is a multifactorial condition involving both embryonic and maternal factors, including impaired endometrial receptivity, immune dysregulation, and broader environmental and lifestyle influences [2,3]. Among embryonic factors, aneuploidy is a major cause of implantation failure [4].

Embryonic aneuploidy, defined as an abnormal chromosome number, increases sharply with advancing maternal age, largely reflecting age-related deterioration in oocyte quality and meiotic fidelity [5]. Preimplantation genetic testing for aneuploidy (PGT-A) enables comprehensive chromosomal assessment of preimplantation embryos and is used to prioritize euploid embryos for transfer [6,7]. In theory, reducing the transfer of aneuploid embryos may improve implantation rates and decrease the risk of miscarriage; however, the clinical utility of PGT-A in women with RIF remains controversial. Some studies have suggested that a greater number of previous implantation failures (IFs) is associated with higher aneuploidy rates [8], whereas others have found no such association, implying that factors beyond embryo ploidy may play a more dominant role in RIF [9]. Furthermore, even after PGT-A, some patients with RIF continue to experience implantation failure following euploid embryo transfer, raising the possibility of persistent endometrial dysfunction or embryo–endometrium asynchrony.

Clinical indications for PGT-A have broadened in recent years to include advanced maternal age (≥38 years) [10], recurrent pregnancy loss [11], and RIF [12]. However, heterogeneity in the definition of RIF contributes to inconsistencies in patient selection and outcome comparisons across studies. To reduce this variability, we adopted the widely cited criteria proposed by Coughlan et al., which define RIF as the failure to achieve a clinical pregnancy in women younger than 40 years despite transfer of at least four good-quality embryos over three or more treatment cycles [13]. International consensus statements have further emphasized the use of age-adjusted thresholds to account for the strong effect of maternal age on reproductive outcomes [14]. Although the association between maternal age and embryo aneuploidy has been well established [15], systematic evaluation of whether the number of previous implantation failures (IFs) is independently associated with embryo ploidy and subsequent pregnancy outcomes in RIF patients undergoing PGT-A remains limited.

Therefore, this retrospective cohort study aimed to investigate whether the number of previous IFs was associated with embryo ploidy status and clinical outcomes after PGT-A in patients with RIF, as defined by Coughlan criteria, with particular emphasis on the potential modifying effect of maternal age.

## 2. Materials and Methods

### 2.1. Study Design and Participants

Between 2017 and 2022, we conducted a retrospective cohort study including 422 women with RIF who underwent their first PGT-A cycle at our center. RIF was defined according to the criteria proposed by Coughlan et al. [13]. Eligible participants were women younger than 40 years who failed to achieve a clinical pregnancy despite the transfer of at least four good-quality embryos across three or more fresh and/or frozen transfer cycles. Exclusion criteria included the use of donor gametes, parental karyotype abnormalities (excluding chromosome polymorphisms), uterine anatomical abnormalities, autoimmune disease (e.g., antiphospholipid syndrome), and major endocrine disorders (e.g., hyperprolactinemia and hyperthyroidism). Ethical approval for this study was obtained from the Institutional Review Board of our center.

### 2.2. Ovulation Induction, Embryo Culture, and PGT-A Workflow

Controlled ovarian hyperstimulation protocols were individualized for each patient according to age, ovarian reserve, and prior response to gonadotropins, in line with routine clinical practice. Final oocyte maturation was triggered with human chorionic gonadotropin (hCG) when at least two follicles reached a mean diameter of ≥18 mm, and oocyte retrieval was performed 34–36 h later. Metaphase II oocytes were fertilized by intracytoplasmic sperm injection, and the resulting embryos were cultured to the blastocyst stage. Trophectoderm biopsy was performed on morphologically suitable day 5 or day 6 blastocysts, and chromosomal copy-number status was determined using next-generation sequencing (NGS). Chromosomal results were reviewed and confirmed by experienced technical staff at our center. According to the laboratory’s predefined reporting criteria during the study period, embryos were classified on the basis of NGS-derived copy-number profiles. Embryos with non-integer chromosomal copy-number results and an estimated abnormal cell proportion of 30–70% were classified as mosaic, whereas those with an estimated abnormal cell proportion of <30% or >70% were classified as euploid and aneuploid, respectively. Endometrial preparation for frozen embryos was individualized and included natural, artificial, or ovulation induction cycles. In frozen embryo transfer cycles, a single euploid blastocyst was transferred. Luteal support was administered according to routine clinical practice.

### 2.3. Outcomes

The primary embryologic outcome was assessed at the embryo level and consisted of the distribution of euploid, aneuploid, and mosaic embryos. A complementary patient-level outcome was the failure to obtain any euploid embryo after PGT-A. Clinical outcomes were evaluated at the single euploid embryo transfer cycle among women who underwent transfer of a single euploid blastocyst and included biochemical pregnancy, clinical pregnancy, ongoing pregnancy, live birth, pregnancy loss, and neonatal birth weight. Biochemical pregnancy was defined as a serum β-hCG concentration of ≥25 mIU/mL measured 12–14 days after embryo transfer. Clinical pregnancy was defined by ultrasonographic confirmation of at least one intrauterine gestational sac with a fetal pole. Ongoing pregnancy was defined as a pregnancy continuing beyond 20 weeks of gestation. Live birth was defined as the delivery of one or more viable infants at a gestational age of ≥28 weeks. Pregnancy losses occurring at ≤12 weeks were classified as early pregnancy loss, whereas those occurring between 12 and 28 weeks were classified as late pregnancy loss.

### 2.4. Statistical Analysis

In this study, the number of previous IFs was used as a categorized measure of failure burden for subgroup stratification (3, 4, or ≥5 IFs), whereas the number of previous embryo transfer attempts was defined as the total number of embryo transfer cycles performed before the index PGT-A cycle and was treated as a separate continuous covariate in the regression analyses. Given the well-established association between advanced maternal age and embryo aneuploidy/euploidy, analyses were stratified a priori by age using a threshold of 38 years (<38 vs. ≥38 years). This cutoff was chosen because 38 years is a clinically relevant threshold in reproductive counseling and PGT-A-related stratification, reflecting the higher expected burden of embryo aneuploidy in older women. Group differences in categorical variables were assessed using the χ^2^ test or Fisher’s exact test, as appropriate, whereas continuous variables were analyzed using appropriate parametric or nonparametric tests. For live birth after single euploid transfer, binary logistic regression was used to estimate adjusted odds ratios (ORs) and 95% confidence intervals (CIs). Covariates were prespecified a priori on the basis of their clinical relevance to embryo competence and pregnancy outcomes, as well as baseline imbalances observed between groups, including age, body mass index (BMI), prolactin level, endometrial thickness, number of previous embryo transfer attempts, miscarriage history, and live birth history. Given the limited number of transfer cycles and live births in some subgroups, these subgroup-specific regression analyses were considered exploratory, and the corresponding results should be interpreted with caution. A two-sided α level of 0.05 was used to define statistical significance, and all analyses were performed using SPSS version 25.0.

## 3. Results

**Figure 1:**
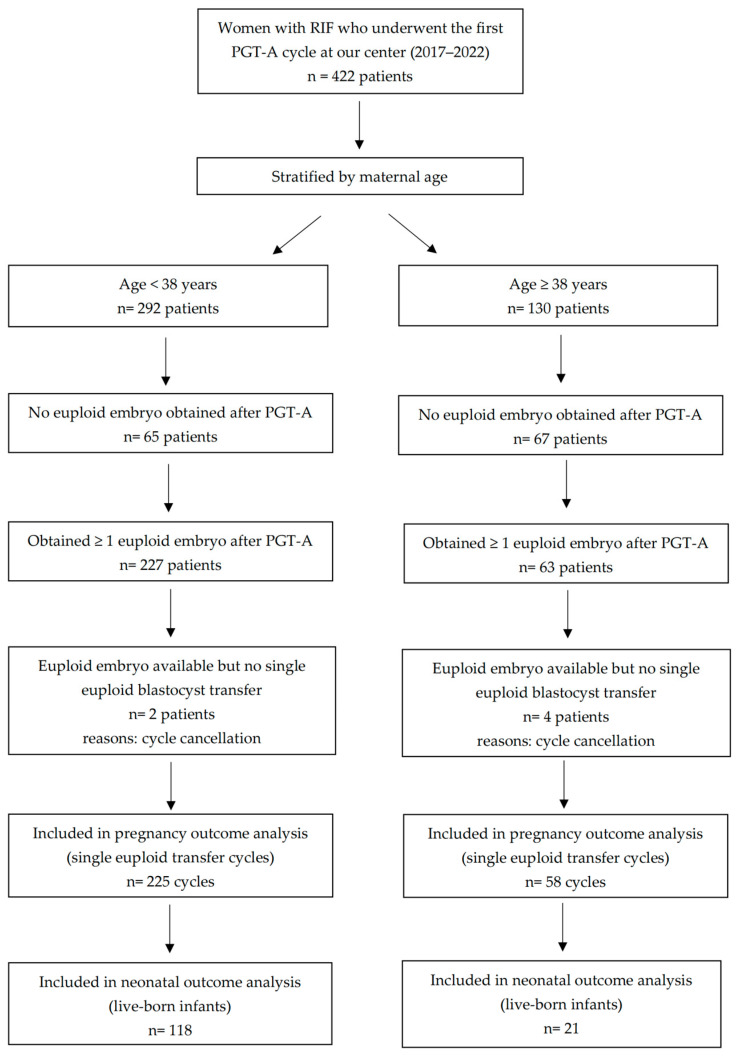
RIF, recurrent implantation failure; PGT-A, preimplantation genetic testing for aneuploidy. The flow diagram shows the derivation of the patient-level, transfer-cycle-level, and neonatal-analysis cohorts. Failure to obtain any euploid embryo after PGT-A was assessed at the patient level; pregnancy outcomes were analyzed per single euploid transfer cycle; neonatal outcomes were derived from live births.

Figure 1 shows the flow of patient selection and the derivation of the patient-level, transfer-cycle-level, and neonatal-analysis cohorts.

### 3.1. Baseline Characteristics of Couples with RIF

A total of 422 women with RIF were stratified by maternal age (<38 years, n = 292; ≥38 years, n = 130) and by the number of previous IFs (3, 4, or ≥5) (Table 1). Baseline characteristics were generally comparable across IF subgroups within each age stratum. Among women aged <38 years, prolactin levels differed modestly across IF categories (*p* = 0.028). The number of previous embryo transfer attempts increased with increasing IF category in both age strata (all *p* < 0.001). Among women aged ≥38 years, miscarriage history and live birth history differed significantly across IF subgroups (*p* = 0.048 and *p* = 0.008, respectively), and endometrial thickness was lower in the ≥5 IF subgroup (*p* = 0.017).

**Table 1:** Characteristics of Patients at Baseline.

Characteristics	<38 Years Old			*p* Value	≥38 Years Old			*p* Value
	3 IFs	4 IFs	≥5 IFs		3 IFs	4 IFs	≥5 IFs	
	(n = 126)	(n = 87)	(n = 79)		(n = 48)	(n = 45)	(n = 37)	
Age, years	34.0 (31.0, 35.0)	32.0 (30.0, 35.0)	33.0 (30.0, 35.0)	0.218	40.0 (39.0, 42.0)	39.0 (39.0, 42.0)	40.0 (39.0, 41.0)	0.747
Body mass index, kg/m^2^	23.59 ± 3.61	22.91 ± 2.82	23.11 ± 2.89	0.268	24.41 ± 3.46	23.92 ± 3.26	23.40 ± 2.45	0.337
Fertility history								
Duration of infertility, years	4.5 (2.5, 6.1)	5.0 (3.0, 6.0)	4.0 (3.0, 6.0)	0.424	3.8 (2.0, 6.9)	5.0 (3.0, 7.5)	4.5 (2.5, 7.8)	0.616
Previous embryo transfer attempts, n	4.91 ± 1.48	6.55 ± 1.72	9.34 ± 2.99	<0.001	4.44 ± 1.11	6.33 ± 2.02	10.30 ± 3.95	<0.001
Previous conception, n/N (%)	75/126 (59.5%)	51/87 (58.6%)	49/79 (62.0%)	0.898	42/48 (87.5%)	31/45 (68.9%)	29/37 (78.4%)	0.093
Previous miscarriage, n/N (%)	70/126 (55.6%)	46/87 (52.9%)	40/79 (50.6%)	0.783	39/48 (72.9%)	26/45 (57.8%)	25/37 (67.6%)	0.048
Previous live birth, n/N (%)	13/126 (10.3%)	12/87 (13.8%)	13/79 (16.5%)	0.431	31/48 (64.6%)	16/45 (35.6%)	14/37 (37.8%)	0.008
Ultrasonographic findings								
Antral follicle count (both ovaries), n	13.0 (9.0, 19.0)	13.0 (10.0, 18.0)	13.0 (10.0, 18.0)	0.462	10.0 (5.3, 13.8)	10.0 (6.0, 12.5)	9.0 (5.5, 14.0)	0.887
Endometrial thickness—cm	0.70 (0.60, 0.90)	0.80 (0.60, 1.00)	0.75 (0.60, 0.90)	0.145	0.75 (0.61, 0.90)	0.80 (0.70, 1.00)	0.65 (0.50, 0.83)	0.017
Laboratory testing								
Anti-Mullerian hormone—ng/mL	2.63 (1.32, 4.92)	2.54 (1.35, 4.54)	2.66 (1.32, 4.02)	0.332	2.05 (1.33, 4.08)	1.46 (1.05, 4.08)	1.99 (1.32, 3.49)	0.647
Follicle-stimulating hormone—IU/L	6.54 (5.54, 7.85)	6.94 (5.75, 8.54)	6.22 (5.43, 7.84)	0.392	6.76 (6.11, 8.82)	7.02 (5.85, 7.83)	6.79 (6.11, 8.87)	0.937
Luteinizing hormone—IU/L	4.69 (3.54, 6.29)	4.95 (3.16, 6.79)	4.37 (3.14, 6.22)	0.774	4.76 (3.62, 6.23)	4.60 (3.51, 6.11)	4.25 (3.07, 5.81)	0.921
Estradiol—pg/mL	35.95 (26.75, 47.48)	37.90 (28.80, 48.40)	39.90 (29.20, 55.80)	0.348	42.40 (32.45, 52.18)	41.20 (29.44, 74.55)	48.50 (29.20, 65.45)	0.111
Total testosterone—ng/dL	20.76 (14.06, 32.93)	22.00 (13.59, 29.35)	22.77 (15.80, 33.01)	0.204	16.93 (10.51, 27.46)	16.94 (11.05, 24.91)	19.70 (13.39, 26.51)	0.828
Prolactin—ng/mL	17.61 (13.01, 32.93)	15.49 (11.59, 20.12)	14.86 (11.48, 21.16)	0.028	14.43 (10.44, 22.78)	14.90 (11.27, 19.77)	13.90 (10.89, 19.32)	0.337
TSH—μIU/mL	2.13 (1.59, 2.80)	2.20 (1.74, 2.79)	2.23 (1.57, 2.85)	0.966	1.94 (1.35, 2.61)	1.97 (1.44, 2.89)	1.86 (1.20, 2.56)	0.360

Note: Baseline steroid hormones were measured on menstrual cycle days 1–3 whenever available, according to routine clinical practice. Continuous variables are presented as mean ± SD or median (IQR), and categorical variables as n (%). IFs, implantation failures; yr, years; BMI, body mass index; AFC, antral follicle count; AMH, anti-Müllerian hormone; FSH, follicle-stimulating hormone; LH, luteinizing hormone; E2, estradiol; TSH, thyroid-stimulating hormone.

### 3.2. Ovarian Stimulation and Embryo Culture Outcomes

Stimulation parameters, oocyte yield, and the number of good-quality day 5/day 6 embryos were comparable across IF categories within each age stratum (Table 2). Similarly, within each age stratum, the distribution of embryo ploidy categories (euploid, aneuploid, and mosaic) did not differ significantly across IF categories (all *p* > 0.05). At the embryo level, the euploidy rate was markedly lower in women aged ≥38 years than in those aged <38 years (24.8% [73/294] vs. 47.3% [465/983]). At the patient level, the proportion of women who failed to obtain any euploid embryo after PGT-A was substantially higher in the ≥38-year group than in the <38-year group (51.5% [67/130] vs. 22.3% [65/292]). Mosaic embryos accounted for 10.9% (32/294) and 19.7% (194/983) of embryos assessed by PGT-A in the ≥38-year and <38-year groups, respectively.

**Table 2:** Outcomes of Controlled Ovarian Hyperstimulation.

Characteristics	<38 Years Old			*p* Value	≥38 Years Old			*p* Value
	3 IFs	4 IFs	≥5 IFs		3 IFs	4 IFs	≥5 IFs	
	(n = 126)	(n = 87)	(n = 79)		(n = 48)	(n = 45)	(n = 37)	
Duration of ovarian stimulation, days	10.0 (8.8, 11.0)	9.0 (8.0, 10.0)	10.0 (9.0, 11.0)	0.139	9.0 (8.0, 11.0)	10.0 (8.0, 11.0)	10.0 (9.0, 11.0)	0.217
Gonadotropin dose-IU	1800.0 (1350.0, 2625.0)	1775.0 (1350.0, 2175.0)	1837.5 (1475.0, 2400.0)	0.107	2150.0 (1475.0, 2400.0)	1837.5 (1475.0, 2400.0)	1837.5 (1475.0, 2400.0)	0.540
Estradiol level on hCG trigger day, pg/mL	2938.00 (2049.75, 4571.00)	2831.50 (2203.75, 4109.00)	3221.00 (1745.50, 5107.00)	0.536	2397.00 (1467.00, 3423.00)	1918.00 (1274.00, 2847.00)	2000.00 (1426.00, 2864.00)	0.558
Endometrial thickness on hCG trigger day—cm	1.00 (0.90, 1.20)	1.00 (0.90, 1.10)	1.00 (0.90, 1.10)	0.813	1.00 (0.85, 1.10)	0.90 (0.83, 1.10)	0.95 (0.80, 1.10)	0.611
Oocytes retrieved, n	10.0 (7.0, 16.0)	10.0 (7.0, 14.0)	12.0 (7.0, 15.0)	0.618	7.0 (5.0, 11.8)	6.0 (5.0, 9.0)	7.0 (4.0, 11.0)	0.865
Good-quality day-5/day-6 embryos, n	3.0 (2.0, 6.0)	3.0 (2.0, 5.0)	3.0 (2.0, 5.0)	0.538	2.0 (1.0, 3.0)	2.0 (1.0, 3.0)	2.0 (1.0, 2.0)	0.945
Embryo ploidy categories after PGT-A, n/N (%)								
Euploid	222/453 (49.0%)	139/280 (49.6%)	104/250 (41.6%)	0.111	30/110 (27.3%)	26/110 (23.6%)	17/74 (22.9%)	0.751
Aneuploidy	131/453 (28.9%)	96/280 (34.3%)	92/250 (36.8%)	0.076	70/110 (63.6%)	70/110 (63.6%)	47/74 (63.5%)	1.000
Mosaic	97/453 (21.4%)	44/280 (15.7%)	53/250 (21.2%)	0.130	10/110 (9.1%)	14/110 (12.7%)	8/74 (10.8%)	0.687

Note: Data are presented as median (IQR) or n/N (%). *p* values compare subgroups by the number of previous implantation failures within each maternal-age stratum (<38 years and ≥38 years). IFs, implantation failures; hCG, human chorionic gonadotropin; IU, international units.

### 3.3. Pregnancy and Neonatal Outcomes Across IF Categories

Among women aged <38 years, pregnancy outcomes were evaluated in 225 single euploid blastocyst transfer cycles. Biochemical pregnancy, clinical pregnancy, ongoing pregnancy, and live birth rates did not differ significantly across IF categories (all *p* > 0.05; Table 3). Clinical pregnancy loss rates were also comparable across IF categories (*p* = 0.773). Among women aged ≥38 years, pregnancy outcomes were evaluated in 58 single euploid blastocyst transfer cycles. No statistically significant differences were observed across IF categories in ongoing pregnancy or live birth rates (all *p* > 0.05), although biochemical and clinical pregnancy rates showed nonsignificant trends (*p* = 0.090 and *p* = 0.081, respectively). Among live births, singleton birth weight and gestational age at delivery were comparable across IF categories in both age strata (Table 3).

**Table 3:** Pregnancy and neonatal outcomes after single euploid blastocyst transfer by previous implantation failure category and maternal age.

Outcome	<38 Years Old			*p* Value	≥38 Years Old			*p* Value
	3 IFs	4 IFs	≥5 IFs		3 IFs	4 IFs	≥5 IFs	
	(n = 103)	(n = 66)	(n = 56)		(n = 23)	(n = 18)	(n = 17)	
Transfer cycles, n	103	66	56		23	18	17	
Biochemical pregnancy, n/N (%)	66/103 (64.1%)	48/66 (72.7%)	39/56 (69.6%)	0.478	12/23 (52.2%)	13/18 (72.2%)	6/17 (35.3%)	0.090
Clinical pregnancy, n/N (%)	58/103 (56.3%)	44/66 (66.7%)	33/56 (58.9%)	0.400	10/23 (43.5%)	12/18 (66.7%)	5/17 (29.4%)	0.081
Ongoing pregnancy, n/N (%)	54/103 (52.4%)	39/66 (59.1%)	29/56 (51.8%)	0.638	9/23 (39.1%)	8/18 (44.4%)	4/17 (23.5%)	0.407
Live-birth, n/N (%)	52/103 (50.5%)	39/66 (59.1%)	27/56 (48.2%)	0.421	9/23 (39.1%)	8/18 (44.4%)	4/17 (23.5%)	0.407
Pregnancy loss								
Biochemical pregnancy loss, n/N (%)	8/66 (12.1%)	4/48 (8.3%)	6/39 (15.4%)	0.593	2/12 (16.7%)	1/13 (7.7%)	1/6 (16.7%)	0.763
Clinical pregnancy loss, n/N (%)	7/66 (10.6%)	6/48 (12.5%)	6/39 (15.4%)	0.773	1/12 (8.3%)	4/13 (30.8%)	1/6 (16.7%)	0.359
First-trimester pregnancy loss, n/N (%)	5/66 (7.6%)	6/48 (12.5%)	4/39 (10.3%)	0.679	1/12 (8.3%)	4/13 (30.8%)	1/6 (16.7%)	0.359
Second-trimester pregnancy loss, n/N (%)	2/66 (3.0%)	0/48 (0)	2/39 (5.1%)	0.316	0/12 (0)	0/13 (0)	0/6 (0)	—
Neonatal characteristics among live births								
Singleton live births, n	50	37	27		9	8	4	
Birth weight of singleton live births, g	3308.4 (2950.0, 3612.5)	3255.7 (3050.0, 3675.0)	3169.2 (2900.0, 3520.0)	0.624	2951.1 (2580.0, 3300.0)	3087.5 (2775.0, 3387.5)	3275.0 (3137.5, 3412.5)	0.400
Twin live births, n	2	2	0		0	0	0	
Birth weight of twin live births, g	2200.0 (2100.0, 2300.0)	3450.0 (3200.0, 3700.0)	0	0.108				
Gestational age at delivery, weeks	38.3 (37.0, 39.0)	38.4 (38.0, 39.0)	38.0 (38.5, 39.0)	0.690	37.4 (36.5, 39.0)	37.5 (36.0, 39.0)	38.3 (37.3, 39.0)	0.654

Note: Data are presented as median (IQR) or n/N (%). Pregnancy outcomes are calculated per transfer cycle unless otherwise specified; pregnancy loss outcomes are calculated per achieved pregnancy (biochemical loss per biochemical pregnancy; clinical/trimester loss per clinical pregnancy). IFs, implantation failures.

**Figure 2:**
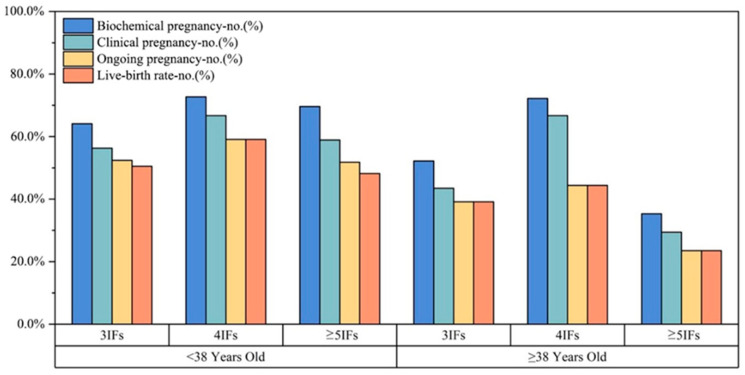
Pregnancy outcomes per single euploid blastocyst transfer cycle stratified by maternal age (<38 vs. ≥38 years) and number of previous implantation failures (3, 4, or ≥5). Bars indicate biochemical pregnancy, clinical pregnancy, ongoing pregnancy, and live birth rates. Numbers of transfer cycles were 103/66/56 for women <38 years and 23/18/17 for women ≥38 years (3, 4, and ≥5 IFs, respectively). Between-group comparisons were not statistically significant within either age stratum; *p* values are shown in Table 3.

Figure 2 shows the biochemical pregnancy, clinical pregnancy, ongoing pregnancy, and live birth rates per transfer cycle across IF categories within each maternal age stratum. Across all subgroups, the rates declined progressively from biochemical pregnancy to live birth. No statistically significant differences were observed between IF categories within either age stratum (Table 3).

### 3.4. Logistic Regression Analysis of Live Birth After Single Euploid Transfer

Multivariable logistic regression models for live birth after single euploid blastocyst transfer were fitted within each IF category (3, 4, and ≥5) and stratified by maternal age (<38 and ≥38 years). After adjustment for age, BMI, number of previous embryo transfer attempts, miscarriage history, live birth history, endometrial thickness, and prolactin, no statistically significant associations with live birth were identified across IF categories in women aged ≥38 years (Table 4) or in women aged <38 years with 3 or 4 IFs (Table 5). In women aged <38 years with ≥5 IFs, a greater number of previous embryo transfer attempts was associated with higher odds of live birth (aOR = 1.258, 95% CI: 1.051–1.505; *p* = 0.012) (Table 5).

**Table 4:** Logistic regression for live birth in women ≥38 years.

Characteristics		3 IFs		4 IFs		≥5 IFs
	*p*	Adjusted OR (95%CI)	*p*	Adjusted OR (95%CI)	*p*	Adjusted OR (95%CI)
Age, years	0.438	0.814 (0.484~1.369)	0.202	0.584 (0.256~1.335)	0.097	0.089 (0.005~1.544)
BMI, kg/m^2^	0.524	1.077 (0.857~1.354)	0.085	0.624 (0.365~1.067)	0.101	2.314 (0.849~6.309
Previous embryo transfer attempts, n	0.237	0.601 (0.258~1.398)	0.656	1.138 (0.643~2.015)	0.179	1.332 (0.877~2.024)
Previous miscarriage	0.737	0.656 (0.056~7.677)	0.082	0.055 (0.002~1.439)	0.127	0.071 (0.002~2.120)
Previous live birth	0.859	0.846 (0.133~5.369)	0.453	0.287 (0.011~7.475)	0.290	0.131 (0.003~5.662)
Endometrial thickness—cm	0.517	0.759 (0.329~1.749)	0.196	3.300 (0.540~20.182)	0.742	1.342 (0.233~7.73)
Prolactin—ng/mL	0.852	1.046 (0.950~1.152)	0.444	1.119 (0.839~1.493)	0.090	0.485 (0.210~1.120)

Note: Outcome was live birth after single euploid blastocyst transfer (live birth = 1). Separate multivariable logistic regression models were fitted within each IF category (3, 4, and ≥5). Data are presented as aOR (95% CI). IFs, implantation failures; aOR, adjusted odds ratio; CI, confidence interval.

**Table 5:** Logistic regression for live birth in women <38 years.

Characteristics		3 IFs		4 IFs		≥5 IFs
	*p*	Adjusted OR (95%CI)	*p*	Adjusted OR (95%CI)	*p*	Adjusted OR (95%CI)
Age, years	0.060	0.883 (0.776~1.005)	0.995	1.000 (0.853~1.173)	0.928	0.992 (0.837~1.176)
BMI, kg/m^2^	0.542	0.969 (0.874~1.073)	0.746	1.027 (0.873~1.209)	0.643	1.044 (0.871~1.25)
Previous embryo transfer attempts, n	0.462	1.101 (0.852~1.422)	0.121	0.807 (0.615~1.059)	0.012	1.258 (1.051~1.505)
Previous miscarriage	0.584	1.230 (0.586~2.579)	0.820	1.113 (0.442~2.799)	0.922	1.054 (0.369~3.005)
Previous live birth	0.383	1.764 (0.493~6.305)	0.317	0.508 (0.135~1.916)	0.155	0.293 (0.054~1.59)
Endometrial thickness—cm	0.164	0.298 (0.054~1.642)	0.421	2.177 (0.327~14.504)	0.446	2.224 (0.285~17.373)
Prolactin—ng/mL	0.924	0.998 (0.962~1.036)	0.893	0.997 (0.955~1.041)	0.261	0.953 (0.877~1.036)

Note: Outcome was live birth after single euploid blastocyst transfer (live birth = 1). Separate multivariable logistic regression models were fitted within each IF category (3, 4, and ≥5). Data are presented as aOR (95% CI). IFs, implantation failures; aOR, adjusted odds ratio; CI, confidence interval.

## 4. Discussion

In this retrospective cohort of women with RIF undergoing PGT-A, the number of previous implantation failures was not independently associated with embryo ploidy or post-transfer clinical outcomes after single euploid blastocyst transfer. By contrast, maternal age showed a clear association with embryo ploidy, with women aged ≥38 years having a substantially lower euploidy rate than those aged <38 years. These findings suggest that, within this RIF population, the cumulative burden of prior implantation failure alone may not adequately reflect embryonic chromosomal competence, whereas maternal age remains a major determinant of euploid embryo yield.

The markedly lower euploidy rate in women aged ≥38 years (24.8% vs. 47.3% in <38 years, *p* < 0.001) is consistent with established evidence on oocyte aging. Mechanistically, maternal age-associated aneuploidy is thought to arise from cumulative defects in meiotic chromosome segregation [16]. These include deterioration of cohesin-mediated sister chromatid cohesion, such as age-related loss of meiosis-specific cohesin including REC8 [17], weakening of spindle assembly checkpoint surveillance [18], and an increased incidence of kinetochore–microtubule attachment errors [19], which collectively promote premature chromatid separation or nondisjunction. In parallel, mitochondrial dysfunction and oxidative stress may impair ATP supply and spindle dynamics, aggravate DNA damage, and ultimately compromise oocyte competence [20]. Together, these molecular alterations provide a plausible biological basis for the substantially reduced euploidy yield observed in older women in our cohort. Consistent with this embryo-level finding, women aged ≥38 years also had a higher patient-level probability of failing to obtain any euploid embryo after PGT-A than women aged <38 years (51.5% [67/130] vs. 22.3% [65/292]).

Although previous IFs were not independently associated with embryo ploidy, women with ≥5 IFs showed features suggestive of a less favorable uterine milieu, including reduced endometrial thickness (6.5 mm vs. 8.0 mm, *p* = 0.017) and a dose-dependent increase in previous embryo transfer attempts (*p* < 0.001). Recurrent implantation failure has been associated with endometrial remodeling characterized by dysregulated local inflammation, impaired angiogenesis, hypoxia-related changes, and fibrotic tendency [21]. From a molecular perspective, disruption of local inflammatory and cytokine networks in the endometrium of patients with RIF may contribute to defective receptivity [22]. Inflammatory mediators such as IL-6 and TNF-α have been reported to be upregulated in RIF and may alter the endometrial microenvironment, thereby affecting early embryo implantation [23,24]. Transcriptomic studies further highlight the complexity of endometrial dysfunction in RIF. Previous work has shown that 53.5% of RIF patients exhibit aberrant expression of endometrial functional genes, including genes related to cell-cycle regulation and ciliary activity [25]. Tan J et al. [26] reported numerically higher pregnancy (66.7% vs. 44.4%) and ongoing pregnancy rates (58.3% vs. 33.3%) with ERA-guided transfer, although these differences did not reach statistical significance (*p* > 0.05). Together, these data suggest that temporal synchronization alone may be insufficient to overcome multifactorial endometrial impairment [27,28].

Consistent with the findings of Cimadomo et al. [9] our analysis indicated that the number of previous implantation failures was not significantly associated with euploidy (OR = 1.12, 95% CI: 0.98–1.28). In the subgroup of women aged <38 years with ≥5 IFs, each additional previous embryo transfer attempt was associated with higher odds of live birth following single euploid blastocyst transfer (aOR = 1.258, 95% CI: 1.051–1.505; *p* = 0.012). However, this subgroup-specific finding should be interpreted with considerable caution. Given the retrospective design, limited subgroup size, and multiple subgroup comparisons, this association may reflect selection factors among patients who proceeded to euploid transfer, survivor bias, unmeasured treatment optimization over time, or residual confounding rather than a true beneficial effect of repeated transfer attempts [29]. Accordingly, this result should be regarded as exploratory and hypothesis-generating rather than evidence that a greater number of prior transfer attempts improves the probability of live birth.

This study provides an integrated perspective on implantation failure in RIF patients undergoing PGT-A by jointly considering embryonic competence and the uterine microenvironment. Several limitations should be acknowledged. First, the retrospective nature and single-center setting may increase susceptibility to selection bias and limit the generalizability of the findings. Second, embryo-level analyses did not account for within-patient clustering, and therefore the embryo-level findings should be interpreted with caution. Third, alternative age parameterizations, including other cutoffs or age as a continuous variable, were not formally evaluated in sensitivity analyses; therefore, the robustness of the chosen age threshold should be interpreted with some caution. Fourth, subgroup-specific regression analyses were based on limited numbers of transfer cycles and live births in some strata and should therefore be considered exploratory. Fifth, multiple subgroup comparisons were performed, increasing the possibility of type I error. Sixth, the absence of a healthy control cohort limits comparisons with normal physiological conditions. Finally, the present study did not directly assess endometrial receptivity, inflammation, fibrosis, or treatment effects. Future multicenter, prospective studies are warranted to validate these findings, clarify possible effect modifiers, and improve risk stratification in RIF patients undergoing PGT-A.

In summary, in this cohort of 422 women with RIF, the number of previous implantation failures was not independently associated with euploidy or live birth after single euploid transfer. Women aged ≥38 years had a markedly lower embryo-level euploidy rate and a substantially higher patient-level probability of failing to obtain any euploid embryo after PGT-A than women aged <38 years. In women aged <38 years with ≥5 IFs, each additional previous embryo transfer attempt was associated with higher odds of live birth, but this subgroup-specific finding should be interpreted cautiously. Overall, these data support a cautious and individualized interpretation of failure burden in RIF and highlight the need for prospective studies integrating both embryonic and endometrial factors.

## 5. Conclusions

Among women with RIF, the number of previous implantation failures was not independently associated with embryo ploidy status or post-transfer outcomes after single euploid blastocyst transfer. Advanced maternal age was strongly associated with reduced euploid embryo yield. In younger patients with ≥5 IFs, the observed association between previous embryo transfer attempts and live birth should be interpreted cautiously, and this subgroup-specific finding is exploratory and requires prospective validation. Overall, these findings support a cautious and individualized interpretation of failure burden in RIF and highlight the need for future studies integrating both embryonic and endometrial factors.

## Data Availability

The individual-level clinical data used in this study are not publicly available because they are derived from hospital patient records and are subject to ethical, institutional, and patient privacy restrictions.
